# The labor market in the digital era: What matters for the Gulf Cooperation Council countries?

**DOI:** 10.3389/fsoc.2022.959091

**Published:** 2022-11-16

**Authors:** Jihen Bousrih, Manal Elhaj, Fatma Hassan

**Affiliations:** Economics Department, College of Business and Administration, Princess Nourah Bint Abdulrahman University, Riyadh, Saudi Arabia

**Keywords:** employment rate, information and communication technologies, digitalization, GCC countries, advanced countries

## Abstract

Digital transformation affects all organizations, large and small. Waves of technological change are frequent and accelerating, requiring constant adaptation by companies and their employees. Artificial intelligence, automation, and digital tools are changing the traditional organizational structure and ways of working. After the COVID-19 pandemic, the labor market has to move toward an inclusive digital transformation that braces the business systems. This paper is an attempt to explore the effect of digitalization on employment in Gulf Cooperation Council (GCC) countries and compare them to some selected advanced countries. The methodology focuses on the second-generation unit root tests and the Auto Regressive Distributed Lagged model for the period 2000–2020. The findings show a negative and significant impact of ICT on employment in the industrial and services sectors for GCC countries with a moderate adjustment speed toward the long-run equilibrium. This result is explained by the shortage of skilled workers in GCC countries compared to advanced countries, where the findings show a positive and significant effect of ICT technologies on total employment, especially in the industrial sector. The adjustment speed toward the long run is significantly higher in advanced countries than in GCC countries.

## Introduction

With the COVID-19 outbreak in 2020, the world turned massively toward modern technologies, accelerating a digital transformation that began several decades ago. Many companies have adopted digital-based business models to continue their activities and save some of their revenues. Simultaneously, mobile applications have been developed to monitor the pandemic's evolution. Researchers are increasingly using artificial intelligence (AI) to better understand the virus and accelerate the development of new vaccines. In some countries, internet use increased by 60% shortly after the outbreak (OCDE, 2020), which proves the digital acceleration. Countries are therefore facing a major challenge. Economies are unlikely to return to “pre-COVID” models. The crisis has clarified the potential of digital technologies. Moreover, our daily activities, such as jobs, education, health, public services, and even social interactions, will be more dependent on technology in the future. Many researchers have defined digitalization as a social-technical phenomenon involving the shift to the daily use of technology in life and business. Among them, we cite El Sawy et al. (2016), Legner et al. (2017), Muro et al. (2017), Alt (2018), Clarke (2019), Chapco-Wade (2018), Sandberg et al. (2020), and Corrocher and Ordanini (2002).

In this paper, we refer to digitalization as the use of digital technologies in day-to-day business life. The growing importance of digital technologies and communication infrastructures in individuals' daily lives, especially in doing work tasks, reveals that companies are increasingly tending to place digital strategies at the heart of their action plans. As countries focus on addressing the COVID-19 crisis and organizing the recovery, it is time for the labor market to work toward an inclusive digital transformation that strengthens the business systems.

The effect of the pandemic on the labor market was remarkable (Piroşc et al., 2021). The COVID-19 crisis has reinforced the importance of digital skills and accelerated digital transformation in businesses. The global labor force moves during a brief period to online work and intensive use of new technologies in several fields such as public administration and the health sector.

In such a situation, all companies must ensure that the labor force can consider the automation process as a weapon in market competition (Madakam et al., 2019). Digital skills become a crucial requirement to be competitive in the labor market. With the online working process, employees need to master the working digital tools, interact with others, and be more productive. Having digital knowledge is more likely to be common among people, but mastering digital skills is another story. Nowadays, finding a job requires having advanced digital skills. Various studies concluded a clear correlation between higher salaries and digital abilities.

The continuous improvement of labor market infrastructures in Gulf Cooperation Council (GCC) countries^1^ and their engagement in impressive national revolution strategies such as the Saudi Vision 2030, Qatar National Vision 2030, and Abu Dhabi Economic Vision 2030 will increase the efficiency across all the sectors mostly due to digitalization. A competitive company requires a skilled digital labor force capable of dealing with changes in the information and communication technology (ICT) sector. Nevertheless, the GCC countries suffer from a shortage of skilled workers. According to Stragtegy Report (2022), the number of digital jobs in the GCC is low compared to other countries. We count only 1.7% of digital jobs in the GCC countries and 5.4% on average in European Union countries. Ninety-three percent of the digital GCC workforce are graduates from foreign universities. This is mainly due to some shortages in the GCC education system which is not updated with the technological changes. Indeed, the region's human capital assessment falls significantly behind the world average. UNDP's Arab Human Development Reports and the two Arab World Competitiveness Reports (2002–2003 and 2005) stressed that education, research, and development are weak in the region and that education systems lack relevance through systems focusing on inputs rather than outcomes. This critical shortage “exacerbates other problems associated with importing both foreign workers and technologies” (Davis and Hayashi, 2007).

**Figure 1:**
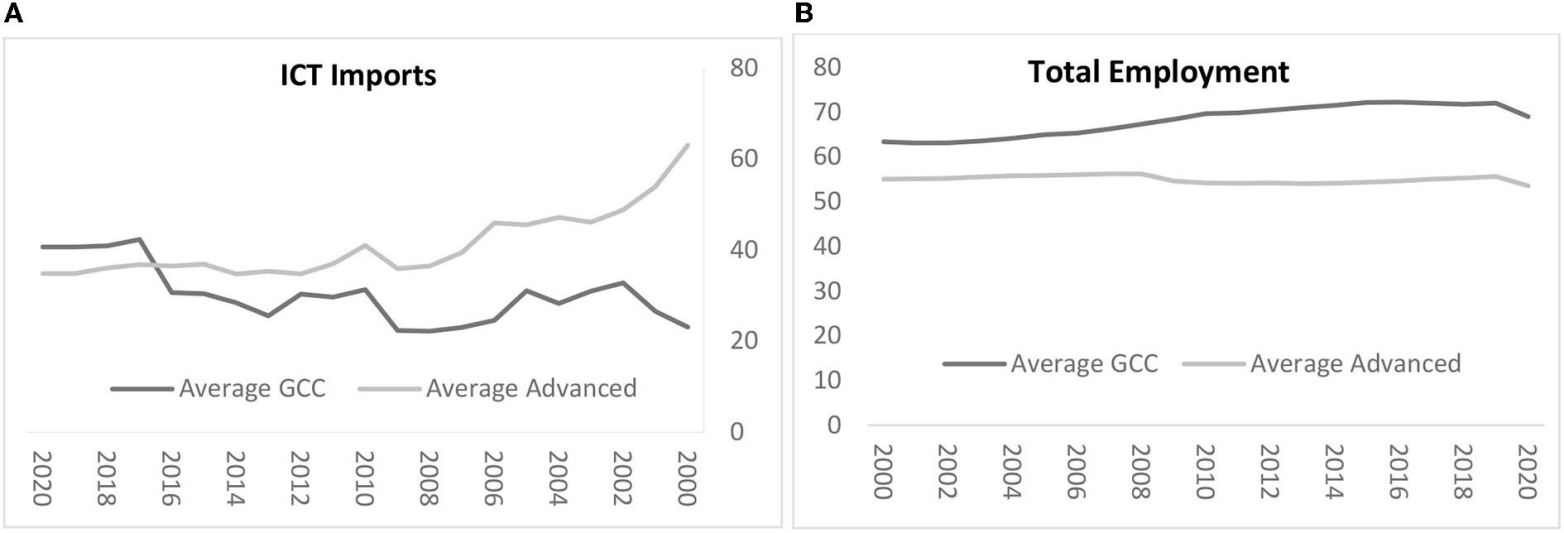
**(A,B)** Digitalization and employment trends in GCC and advanced countries. Source: Authors' calculations based on World Bank Database.

According to Figure 1A, we use ICT imports as a proxy for digitalization in GCC and advanced countries. The digital economic index classifies countries into the following three groups: digital creators, digital adopters, and digital disruptors. The GCC countries belong to the group of digital adopters which makes them dependent on digital creators, through the imports of ICT.

Since 2015 and after the reform plans of Saudi Arabia, Emirates, and Qatar, GCC economies have recorded important digital sector growth to become more competitive in the global market. They can now host strong multinational firms.

Figure 1B shows that the percentage of employed persons is higher in GCC countries than in advanced countries. This is mainly due to the importance of manual jobs that did not require any advanced skills. Thus, companies increase the number of hires to improve productivity. Since 2018, we record a slight decrease in employment which can be explained by the growth of the digital sector which requires fewer nonskilled workers. The shortage of workers with advanced technical skills reduces the employment rate. In fact, the employment rate in GCC countries is calculated using the participation of national workers in the labor force, not foreign workers.

The situation of the GCC countries was controverted in the literature. Therefore, this paper attempts to explore digitalization's impact on labor market indicators. The novelty of this paper is to be an innovative study on the impact of modern technologies use after the COVID-19 outbreak on employment for GCC countries from different perspectives: industrial sector, services sector, and women's participation in the labor force. Moreover, we compare the performance of the GCC countries to some selected advanced countries to find out recommendations to support the GCC job market in the digital era.

The paper is presented as follows: after a concise presentation of the main research problem, the first section introduces the literature review. The second section defines the data and the methodology used in the paper, followed by the empirical results, and finally, in the fourth section, we present the conclusion and the recommendations.

## Literature review

Technology and labor market variables rely on macroeconomic theories, typically the growth models where technology, labor, and capital are the most important key factors that generate economic growth. Several studies confirmed that digitalization fundamentally changes the nature of work. Modern technologies impact the labor market in many aspects such as reducing the routine of automated work and increasing the demand and pay for highly skilled technical workers. The process of work digitalization in the last decades has gone through various stages, and there is no doubt that during the COVID-19 pandemic it was significantly modified and accelerated (Sam, 2020; Janeska and Lozanoska, 2021).

Schilirò's (2021) study, on digital transformations during the COVID-19 pandemic, shows that digital transformation causes several changes in the economy and society and these changes may be beneficial for one and harmful for others. The substitution of workers for machines will produce counterbalancing forces by increasing the number of lost jobs on the one hand and by creating new types of jobs on the other hand.

An empirical paper by Katz et al. (2021) provides various approaches to measure job gains and losses due to automation. Their application in Chile indicated that lost jobs are equal to the jobs created; thereby it results in a slight impact on the labor force. However, the jobs created are mainly addressed to workers with a high degree of education. Their results indicate probable social marginalization impacts, as some weak groups of workers will face the risk of losing their businesses due to their low level of education and income. Another paper by Jongwanich et al. (2022) examined the impact of advanced technology (proxies by ICT) on variations in employment and revenue in Thailand. The results show that advanced technology cannot push workers out of the labor market, but it reallocates the workers between skilled and unskilled positions.

The digital revolution brings new ways of working but cannot be a solution to all the problems. Wisskirchen (2017) argues that the high level of unemployment in some sectors cannot be avoided by the introduction of new technologies, but it will be a shift in some work areas especially in the services sector, by allowing some workers to benefit from flexible work time and workplace through the remote work. However, Denishin (2020) argues that the impact of digitalization is still not clear. Manual jobs have still a high impact on the economy globally and the industrial sector especially. Thus, we cannot think about the alternative of zero manual jobs in the future, but we have to explore the alternative that robots and individuals can work together side by side. These findings are confirmed by Idris et al. (2021) in their research on the effect of high-technology trade on employment. The authors confirmed that there is evidence of a negative relationship between high-tech trade and employment. Illéssy et al. (2021) stated that highly qualified employees are more likely to get a job and stay in their jobs.

Despite the national effects, digitalization reinforces the attractiveness of countries in the international market and helps to improve business practices in the international business world. A paper by Bezrukova et al. (2022) on the effect of digitalization on the expansion of the world economy finds that the European Union member states have developed and implemented digital technologies to reach a high economic expansion and a competitive business environment. Vasilescu et al. (2020) tried to investigate if digitalization leads to creating vulnerable citizens or countries groups by focusing only on their digital skills. The survey of EU citizens (2020) shows that people with a high level of digital skills have a confident perception of the effect of technologies on the economy.

The unemployment rate is one important indicator of the labor market. Controversial studies tried to identify the relationship between digital technology expansion, ICT developments, and unemployment. Among them, Abbasabadi and Soleimani (2021) used the following three indexes of technology: the ICT development index, the digital index, and the technological readiness index. Their findings revealed a clear relationship between unemployment and digital technology indexes for 163 countries. As digital technologies are expanding, unemployment grows to a maximum and then begins to decline as technology expansion exceeds a specific value. Another study by Neffati and Gouider (2019) analyzed the socioeconomic effects of digitization in the Kingdom of Saudi Arabia by using data covering the period from 1981 to 2016. Their results show a significant negative relationship between digitalization and unemployment. These findings are confirmed by Başol and Yalçin (2021) and Ivanitskaia (2022).

For the GCC countries, the Ideation Center Insight Report (2017) demonstrates that GCC countries are engaged in national transformation plans such as the 2030 vision in the Kingdom of Saudi Arabia and the United Arab Emirates to increase efficiency across all sectors through digitalization. GCC countries still need a comprehensive strategy to focus on boosting digital high skills and developing a more skilled workforce that can adapt easily to modern technologies, besides creating attractive employment chances. The cultural norms and some restrictive gender rules applied in GCC countries are holding women back from joining the workforce. Since 2015, the reform plans applied in Saudi Arabia, United Arab Emirates, and Qatar, as well as new policies are encouraging women to join the workforce and increase their visibility in the labor market. Gadi (2021) focuses on the obstacles to women's participation in the GCC labor market and the author shows that insufficient experience and training in digital technology were the main identified obstacles.

## Data and methodology

### Data

In this section, we define the variables of interest to study the impact of digitalization on labor market indicators. The definition of the variables used in the analysis is recorded in Table 1. The data used in the regression were gathered from the World Bank tables. We selected annual data that cover the period 2000–2020. The selected sample includes six GCC countries as follows: Kingdom of Saudi Arabia, United Arab Emirates, Qatar, Bahrain, Kuwait, and Oman, as well as the following six advanced countries: the United States, Canada, the United Kingdom, France, Italy, and Germany. The analysis was performed using EVIEWS 10.

**Table 1:** Economic variables included in the model.

**Name**	**Code**	**Definition**
Total employment	Tot-emp	Employment to population ratio is the proportion of a country's population that is employed.
		Unit: 15+ age, total (%)
Industry Employment	Indus-emp	Employment is defined as persons of working age who were engaged in any activity to produce goods or provide services for pay or profit. The industry sector consists of mining and quarrying, manufacturing, construction, and public utilities (electricity, gas, and water).
		Unit: (%) of total employment
Services Employment	Services-emp	Employment is defined as persons of working age who were engaged in any activity to produce goods or provide services for pay or profit. The services sector consists of wholesale and retail trade and restaurants and hotels; transport, storage, and communications; financing, insurance, real estate, and business services; and community, social, and personal services.
		Unit: (%) of total employment
Women Business and the Law Index	Wom-B	The index measures how laws and regulations affect women's economic opportunities.
		Unit: Scale 1–100
Information and communication technology imports	Ict	Information and communication technology goods imports include computers and peripheral equipment, communication equipment, consumer electronic equipment, electronic components, and other information and technology goods.
		Unit: % total goods imports
High-technology exports	Hightec	High-technology exports are products with high R&D intensity, such as aerospace, computers, pharmaceuticals, scientific instruments, and electrical machinery.
		Unit: current US$
Mobile cellular subscriptions	Mobile	Mobile cellular telephone subscriptions are subscriptions to a public mobile telephone service that provides access to the PSTN using cellular technology.
		Unit: per unit
Trademark applications	Tram	Trademark applications filed are applications to register a trademark with a national or regional Intellectual Property (IP) office.
		Unit: per unit

World Bank (2022).

### Methodology

This research is an attempt to study the effect of digitalization on total employment and different sectors in GCC countries and some selected advanced countries. We apply a comparable model to that of Shahbaz and Rahman (2012) and Dinh et al. (2019), and the model is stated as follows:


(1) $${\text{Y}}_{\text{it}}=\alpha +\;\beta {\text{X}}_{\text{it}}+\varepsilon_{\text{it}}$$

where ***Y_it_*** is the endogenous variable, **α** is the intercept, and β is the partial coefficient for the exogenous variables ***X_it_*** (ICT, Mobile, and Tram).

## Empirical results

### Descriptive statistics

The descriptive statistics provide quantitative insights into the selected data series. Table 2 below presents the central measures and the standard deviation. The number of observations is different for the two samples because the data for GCC countries start from 2001 to 2020, while it starts from 2000 to 2020 for the selected advanced countries. The results show a positive mean of all the selected variables over the study period. Yet, a high standard deviation presents in mobile and trademark compared to the other variables used in the model. These findings are similar for the GCC and the advanced countries.

**Table 2:** Descriptive statistics.

	**Total employ**	**Employ industry**	**Employ service**	**ICT**	**Mobile**	**Trademark**	**Women business**
GCC countries
Mean	60.50250	39.47242	65.34550	5.411476	9143000	10426.97	30.51042
Median	67.34500	34.95500	64.13000	4.901080	3481466.	8976.000	29.37500
Maximum	87.75000	76.80000	82.42000	17.18817	54000000	37669.00	70.62500
Minimum	20.45000	11.19000	38.86000	0.000906	120856.0	1000.000	23.75000
Std. Dev.	20.75441	17.41391	9.832287	2.581175	13967351	6178.703	6.054171
Skewness	−0.689246	0.799247	−0.781310	1.287784	2.188330	1.493783	3.531333
Kurtosis	2.345810	2.619924	3.109731	6.193084	6.656599	6.924595	21.41987
Jarque-Bera	11.64103	13.49820	12.26910	84.14669	162.6294	121.6400	1945.865
Probability	0.002966	0.001172	0.002167	0.000000	0.000000	0.000000	0.000000
Sum	7260.300	4736.690	7841.460	649.3771	1.10E+09	1251236.	3661.250
Sum Sq. Dev.	51258.71	36086.05	11504.19	792.8335	2.32E+16	4.54E+09	4361.706
Observations	120	120	120	120	120	120	120
Advanced countries
Mean	55.03008	23.85857	73.87563	9.221709	1.01E+08	105331.5	91.59722
Median	57.36500	22.72000	74.92500	8.391329	76605440	69215.00	94.37500
Maximum	63.77000	33.53000	80.83000	18.67351	4.42E+08	492729.0	100.0000
Minimum	42.54000	18.12000	62.98000	4.806316	8727000.	33846.00	76.87500
Std. Dev.	6.215070	4.143330	4.856365	3.077355	96017044	106570.2	6.569130
Skewness	−0.624443	0.546795	−0.537165	0.728972	2.168463	2.117852	−0.616904
Kurtosis	2.117341	2.111128	2.201606	2.752706	7.020183	6.598867	2.124960
Jarque–Bera	12.27871	10.42667	9.405992	11.48045	183.5967	162.1884	12.01189
Probability	0.002156	0.005443	0.009068	0.003214	0.000000	0.000000	0.002464
Sum	6933.790	3006.180	9308.330	1161.935	1.27E+10	13271763	11541.25
Sum Sq. Dev.	4828.386	2145.897	2948.036	1183.764	1.15E+18	1.42E+12	5394.184
Observations	126	126	126	126	126	126	126

## Correlation matrix

To investigate the possible correlation between the variables of interest, the correlation matrix is presented in Table 3. The results show that, for advanced countries, there is no high correlation between the variables. For the advanced countries, there is a high and positive correlation between trademark applications and mobile subscriptions with 0.8654.

**Table 3:** Correlation matrix.

	**ICT**	**Mobile**	**Tram**	**Empind**	**Empserv**	**Emp-Tot**	**Wom-B**
	GCC countries
ICT	1	0.5660	0.7085	−0.3387	0.4425	0.6566	−0.5897
Mobile		1	0.7736	0.0303	0.0084	−0.0285	−0.7379
Tram			1	−0.3483	0.3846	0.3283	−0.5212
Empind				1	−0.9806	−0.5727	−0.1327
Empserv					1	0.6976	0.1110
Emp-Tot						1	−0.0107
Wom-B							1
	Advanced countries
ICT	1	0.3465	0.3823	−0.0679	0.3014	−0.2189	−0.0511
Mobile		1	0.8654	−0.5778	0.5018	−0.1531	−0.0673
Tram			1	−0.4213	0.5156	−0.1794	0.0062
Empind				1	−0.2808	−0.3985	−0.2080
Empserv					1	−0.6831	0.0478
Emp-Tot						1	0.1433
Wom-B							1

### Cross-sectional dependence test

To test the robustness of the model, we proceed to investigate the cross-sectional dependence. We apply Breusch and Pagan's (1980) LM test, Pesaran's (2004) scaled LM test, and Pesaran's (2004) CD test. The findings in Table 4 show that the null hypothesis of “no cross-sectional dependence” is rejected at a 1, 5, and 10% significance level for all the models except Model 4 for GCC countries. Thus, the findings confirm the presence of cross-sectional dependence among the variables.

**Table 4:** Cross-sectional dependence test.

	**Breusch–Pagan LM**	**Pesaran scaled LM**	**Pesaran CD**
	GCC countries		
Model 1	43.0243 (0.0002)	5.1165 (0.0000)	2.07335 (0.0381)
Model 2	50.5148 (0.0000)	6.4840 (0.0000)	0.9837 (0.3252)
Model 3	90.6647 (0.0000)	13.8144 (0.0000)	8.6275 (0.0000)
Model 4	34.7184 (0.0027)	3.6000 (0.0003)	1.0889 (0.2762)
	Advanced countries		
Model 1	175.7841 (0.0000)	29.3550 (0.0000)	12.7038 (0.0000)
Model 2	47.9313 (0.0000)	6.0124 (0.0000)	3.4984 (0.0000)
Model 3	187.0221 (0.0000)	31.406 (0.0000)	13.2199 (0.0000)
Model 4	182.4419 (0.0000)	30.5705 (0.0000)	12.9138 (0.0000)

Probabilities are reported in brackets.

### Unit root tests

The presence of cross-sectional dependence among the models reduces the efficiency of the first-generation unit root tests. Thus, to check the stationarity of the time series and to determine the order of integration of the data we use the second-generation unit root tests: cross-section augmented Dickey–Fuller (CADF) and Im, Pesaran, and Shin (CIPS) unit root tests using Akaike information criterion (AIC). According to Hill et al. (2011), stationarity is a prior condition to any regression. All variables should be stationary to prevent any statistical problems in the regression. The CADF equation is as follows:


(2) $$\begin{array}{l} \Delta {\text{Y}}_{\text{i}\;\text{t}}=\;\theta_{\text{i}\;\text{t}}\gamma_{\text{i}}+\;\rho_{\text{i}}{\text{Y}}_{\text{i}\;\text{t}-1}+\sum_{\text{L}=1}^{\text{pi}}\vartheta_{\text{i}\;\text{L}}\Delta {\text{Y}}_{\text{i}\;\text{t}-\text{L}}\\ \;+\alpha_{\text{i}}{\bar{Y}}_{\text{t}-1}+\sum_{\text{L}=0}^{\text{pi}}\varphi_{\text{i}\;\text{L}}\Delta {\bar{Y}}_{\text{t}-\text{L}}+\varepsilon_{\text{i}\;\text{t}} \end{array}$$

where Ȳ_*t*_ is the cross-section average of *Y*_*it*_.


(3) $$\text{CIPS}\;=\;{\text{n}}^{-1}\sum_{\text{i}=1}^{\text{n}}\text{t}{\text{r}}_{\text{i}}$$

where tr is the t ratio on the coefficient in Equation 2 above. The critical value of this t ratio is tabulated by Pesaran (2007).

The stationarity tests were performed on the level and first difference to examine the order of integration of each variable. Table 5 presents the results of the CADF and CIPS unit root tests. The findings allow us to reject the null hypothesis of the presence of a unit root among the variables.

**Table 5:** Second-generation unit root tests.

**Variables**	**CIPS**		**CADF**	
	**Level**	**First**	**Level**	**First**
		**difference**		**difference**
GCC countries				
Emp Tot	−2.166	−2.464**	−2.166	−2.464**
Emp Ind	−2.753***	−3.944***	−2.565**	−2.847***
Emp serv	−2.258*	−3.110***	−2.573**	−2.992***
ICT	−2.175	−4.787***	−1.558	−2.918***
Mobile	−2.670***	−3.197***	−2.387**	−3.197***
Tram	−1.753	−3.552***	−2.363**	−2.944***
Wom–b	−1.113	−2.760***	−1.494	−2.760***
Advanced countries				
EmpTot	−0.679	−2.572**	−0.679	−2.572**
EmpInd	−2.652***	−4.236***	−2.678***	−2.421**
Emp serv	−2.580***	−4.426***	−2.438**	−2.585**
ICT	−3.961***	−5.002***	−2.568**	−3.957***
Mobile	−2.068	−3.328***	−1.540	−2.587**
Tram	−2.127	−4.149***	−1.989	−3.314***
Wom-b	−1.790	−4.620***	−1.106	−3.002***

***, **, and * denote 1, 5, and 10% significant levels, respectively.

For the GCC group, emp industry, emp services, and mobile are stationary in level according to CIPS and CADF tests; cointegrated of order zero I (0), while the remaining variables are cointegrated of order one I (1). For the advanced countries group, ICT, emp services, and emp industry variables are stationary in Level I (0) according to CIPS and CADF tests. For the other variables, we confirm the presence of a unit root and the cointegration of order 1 I (1).

### Cointegration test

The cointegration test sheds light on the number of cointegration relationships and their functional form. We performed the Pedroni Panel cointegration test based on the comparison likelihood ratio to its critical value. The test hypothesis is formulated as follows: H0: There is no cointegration relationship between Mobile, ICT, Tram, and the labor market indicators; H1: There is a cointegration relationship between Mobile, ICT, Tram, and the labor market indicators.

The results are summarized in Table 6 below. We define four models to assess the impact of digitalization on different indicators of the labor market. Model 1 links the digitalization proxies' variables (mobile subscription, trademark, and ICT) to total employment. Model 2 links the same range of independent variables to employment in the industrial sector, Model 3 explores the impact of these variables on employment in the services sector, and Model 4 links the employment of women to digitalization. We apply these four models to GCC countries and advanced countries to identify the differences between these economies. According to Table 6, the findings of Pedroni's cointegration tests are shown which confirm the absence of a long-run association among the proposed variables.

**Table 6:** Pedroni residual cointegration tests.

	**Model 1**	**Model 2**	**Model 3**	**Model 4**
	GCC countries
H1: Common coefficients (within dimensions)
P v-statistic	−1.172113	0.633885	−0.101939	0.896599
P rho-statistic	1.520516	0.290873	0.383327	−0.226090
P pp-statistic	−0.021423	−5.886722***	−7.805899***	−3.523490***
P ADF-statistic	0.785963	−2.594481**	−3.396892***	−0.254567
W v-statistic	−0.821049	0.302369	0.272527	0.149517
W rho-statistic	1.289937	0.373631	−0.109908	0.338533
W pp-statistic	−0.592470	−2.926552**	−4.658678***	−2.396348**
W ADF-statistic	0.732168	−0.268991	−1.042643	0.444498
H1: Individual coefficients (between dimensions)
Group rho-statistic	1.821381	1.282283	0.739910	1.106013
Group pp-statistic	−1.174652	−3.240137***	−5.850178***	−2.369402**
Group ADF- statistic	1.085788	0.200126	−0.540473	1.026846
	Advanced countries
H1: Common coefficients (within dimensions)
P v-statistic	2.169380	−0.327763	−0.437480	0.350512
P rho-statistic	0.916764	0.577961	0.563019	1.005945
P pp-statistic	0.292567	−2.202060**	−2.263254**	−1.629248*
P ADF-statistic	−0.175122	−1.759037**	−2.009502**	−2.269875**
W v-statistic	0.678470	−1.094061	−1.059896	−1.844610
W rho-statistic	1.305321	1.280154	1.319228	1.765851
W pp-statistic	0.466770	−1.1321110	−1.016586	−0.975898
W ADF-statistic	0.277070	−2.473375**	−2.800741***	−4.284281***
H1: Individual coefficients (between dimensions)
Group rho-statistic	1.887132	2.187693	2.091417	2.205137
Group pp-statistic	1.108554	−1.051130	−1.159915	−1.142548
Group ADF- statistic	0.234244	−2.301980**	−2.610696***	−2.987539***

***, **, and * denote 1, 5, and 10% significant levels, respectively.

### Panel autoregressive distributed lag (Panel ARDL) model

Due to the presence of different levels of integration among time series variables, we use the Panel ARDL approach rather than the traditional estimation approach. Panel ARDL methods are characterized by ranges of benefits that permit estimating different variables with different orders of stationarity as expressed in Table 5.

The Panel ARDL model is very useful when investigating the short and long-run relationship between digitalization and labor market indicators (Pedroni, 2004), it can be formulated as follows:


(4) $$\begin{array}{l} y_{i,t}=\alpha_{i}+\delta_{i}y_{i,t-1}+\beta_{i}x_{i,t}+\overset{p}{\underset{j=1}{\sum }}{\gamma_{ij}y}_{it-j}+\overset{q}{\underset{j=0}{\sum }}{\vartheta_{ij}X}_{ij}+\mu_{it}\; \end{array}$$

where the parameter δ_*i*_ captures adjustment toward long-run equilibrium, *p, q* is the optimal lag length determined by the AIC criterion. The endogenous variable *y*_*t*_ represents different indicators from the labor market, and the exogenous variable *X*_*t*_ represents ICT, mobile subscription, and trademark applications.

The results of the Panel ARDL model are summarized in Table 7 below. According to the first part (a) referring to GCC countries, the long-run relation between employment in the industrial sector and the variables of interest (Model 2), shows that Mobile and Tram positively affected industry employment at a 1% significance level, while ICT has a significant negative impact on industry employment at 1%. This result can be explained by the importance of the oil industry in the Gulf region. The petroleum leading country in the region is the Kingdom of Saudi Arabia. Since 2016, the country, and especially ARAMCO is engaged in many digital transformations to meet the world's energy needs. The Emirates with ADNOC company are shifting from a traditional oil company to an innovative international energy company in the era of oil and Gas 4.0. Thus, the new automatization of the oil industry in the region increased the unemployment in this sector due to a shortage of national skilled workers. These results for GCC countries are confirmed by Al Qudah et al. (2016) and Wisskirchen (2017). The second interesting result for the GCC countries is the negative long-run coefficient for a mobile subscription that negatively affects employment in the services sector. Many GCC countries, since the COVID-19 pandemic provided an online portal that operates as an entry point to digital public services. Since that time, people become more familiar with e-services, and they do not need any more to visit governmental entities to apply for public services so this impact negatively the employment rate in the sector. One can cite various public applications such as TAWAKALNA for Saudi Arabia, TAMM for Emirates, and TAWASUL for Bahrain. Indeed, GCC governments continue to improve and innovate in the public sector to be competitive compared to other countries.

**Table 7:** Panel ARDL results.

	**Model 1**	**Model 2**	**Model 3**	**Model 4**
	GCC countries (a)
	Long-run Equation
Ict	−0.000600	−0.0049440***	−0.015521	−2.561476
Mobile	0.1093993***	0.046475***	−0.405479***	0.270937
Tram	−0.137944***	0.106300***	0.793005***	2.059094
	Short-run equation
CointeQ01	−0.131353	−0.300004**	−0.88190	−0.076496
D [Dependant variable (-1)]	0.336958***	0.203298	0.165236	1.189479
D (ict)	0.001714	−0.008856	−0.00326	0.150450
D (Mobile)	−0.011846	−0.004859	−0.012023	−0.097120
D (Tram)	0.008838	0.001553	−0.005784	−0.149204
AIC criteria	−5.751198	−3.917388	−5.314600	−3.166857
Schwarz criteria	−4.427140	−3.011453	−3.990541	−1.842798
Log Likelihood	402.0719	274.0433	375.8760	247.0114
	Advanced countries (b)
	Long-run Equation
Ict	0.159112***	0.275712***	−0.095697***	−0.374650***
Mobile	0.082525***	−0.065814*	0.008114	0.085861
Tram	−0.0113448***	−0.073502***	0.012615***	0.198459**
	Short-run equation
CointeQ01	−0.289455***	−0.358416***	−0.263254***	−0.202385*
D [Dependant variable(−1)]	0.452032***	−0.065845**	−0.008722	
D (ict)	−0.050052	−0.118144***	0.028629***	−0.003235
D (Mobile)	−0.046554	−0.063591	0.010265	0.092303
D (Tram)	0.113003***	0.038430	−0.001031	−0.011340
AIC criteria	−5.555948	−5.194752	−7.185032	−5.357264
Schwarz criteria	−4.272868	−4.046733	−6.307136	−4.614429
Log Likelihood	407.0247	378.2694	491.6570	370.5076

***, **, and * denote 1, 5, and 10% significant levels, respectively.

Model 4 results show that Mobile and Tram positively affect women's employment, but the results are not significant. Thus, we cannot conclude a clear relationship between these variables. Indeed, in the GCC countries, women's participation in the labor market and the number of business owners were lower compared to men before 2015. The new reform plans adopted after 2015, especially in Saudi Arabia are likely to increase women's involvement in the labor market, Elhaj and Pawar (2019).

For the advanced countries group, the results of Model 1 show that ICT and Mobile affect positively total employment at a 1% level of significance. Model 2 shows a positive relation between employment in the industrial sector and ICT, the relationship is significant at a 1% level. Regarding employment in the services sector, only the variable Tram has a positive and significant impact. Finally, the results present a positive impact of Mobile and Tram on the integration of businesswomen in the labor market. These results are explained by the important proportion of national skilled workers in the labor market. This is not the case for the GCC countries where an important number of skilled expatriates' workers are dominating the digital labor force compared to the national skilled workers. This gap is mainly due to the mismatching of the educational and training systems in these countries with the job market requirements which impacts the employment rate.

Considering the short-run equation (Table 7), the coefficient cointeQ01 shows the error correction mechanism ECM, which explores the speed of convergences to equilibrium from the short run to the long run per 1 year. The sign of this coefficient is negative and significant at 1% for GCC Model 2 when industry employment was the dependent variable. It shows a 30.0004% adjustment in industry employment from the short run to the long run annually. In other words, to achieve long-run equilibrium, the GCCs will need 3.33 years. For the Advanced countries group, all the models show the significance of the error correction mechanism ECM, with respectively 28.9455% for Model 1; 35.8416% for Model 2; 26.3254% for Model 3, and 20.2385% for Model 4. Therefore, the advanced countries will achieve the long-run equilibrium faster than the GCC countries for employment in the industrial sector with 2.79 years and will record a slower adjustment speed for total employment, employment in the services sector, and the employment of women with 3.45, 3.79, and 4.94 years, respectively.

### Dumitreu and hurlin causality test

Finally, the Dumitrescu and Hurlin (2012) panel causality test was run to test the causality among the variables. Dumitreu and Hurlin's test is based on the VAR model and assumes that there is no cross-sectional dependency. However, the simulations by Monte Carlo show that even under the conditions of cross-sectional dependency, this test can produce strong findings.


(5) $$\begin{array}{l} Y_{i,\;t}=\;c_{i\;}+\sum_{k=1}^{pi}\gamma_{i\;K}\text{Δ}Y_{i,\;t-K}+\sum_{k=1}^{pi}\beta_{i\;K}X_{i,\;t-K}\\ \;+\varepsilon_{i\;t},i=1,\ldots ,N\;and\;t=1,\ldots T \end{array}$$

where *P* is the lag length, β_*i K*_ and δ_*i K*_ are the short-run coefficients.

The results are presented in Table 8 below. The causality test results show a causal and significant relation between the selected variables for the two samples.

**Table 8:** Dumitreu and hurlin causality test results.

**Null hypothesis**	**GCCs**		**Advanced countries**	
**Model 1**	**W-Stat**	**Zbar-Stat**.	**W-Stat**	**Zbar-Stat**.
ICT does not homogeneously cause EMP_TOT EMP_TOT does not homogeneously cause ICT	2.53556 3.61231	0.14825 1.07670	4.38097* 2.02184	1.81672 −0.27636
MOBILE does not homogeneously cause EMP_ TOT EMP_TOT does not homogeneously cause MOBILE	2.84982 6.44276***	0.41922 3.51731	4.49135* 2.19106	1.91465 −0.12623
TRAM does not homogeneously cause EMP_TOT EMP_TOT does not homogeneously cause TRAM	3.81068 4.45778*	1.24774 1.80572	5.83427*** 5.38442**	3.10613 2.70701
MOBILE does not homogeneously cause ICT ICT does not homogeneously cause MOBILE	3.17187 6.59657***	0.69692 3.64994	7.83305*** 2.00758	4.87950 −0.28902
TRAM does not homogeneously cause ICT ICT does not homogeneously cause TRAM	3.35374 1.32101	0.85374 −0.89903	2.83679 3.63561	0.44668 1.15542
TRAM does not homogeneously cause MOBILE MOBILE does not homogeneously cause TRAM	4.77790** 3.75382	2.08176 1.19872	2.32233 3.47835	−0.00976 1.01589
Model 2				
EMPIND does not homogeneously cause ICT ICT does not homogeneously cause EMPIND	3.82015 1.80733	1.25591 −0.47969	7.51662*** 6.94062***	4.59875 4.08771
EMPIND does not homogeneously cause MOBILE MOBILE does not homogeneously cause EMPIND	2.87990 2.38639	0.44516 0.01962	3.72631 5.39252**	1.23589 2.71419
EMPIND does not homogeneously cause TRAM TRAM does not homogeneously cause EMPIND	5.82699*** 6.89555***	2.98636 3.90775	2.03566 1.56874	−0.26410 −0.67837
MOBILE does not homogeneously cause ICT ICT does not homogeneously cause MOBILE	3.17187 6.59657***	0.69692 3.64994	7.83305*** 2.00758	4.87950 −0.28902
TRAM does not homogeneously cause ICT ICT does not homogeneously cause TRAM	3.35374 1.32101	0.85374 −0.89903	2.83679 3.63561	0.44668 1.15542
TRAM does not homogeneously cause MOBILE MOBILE does not homogeneously cause TRAM	4.77790** 3.75382	2.08176 1.19872	2.32233 3.47835	−0.00976 1.01589
Model 3				
EMPSERV does not homogeneously cause ICT ICT does not homogeneously cause EMPSERV	2.87797 3.20682	0.44349 0.72706	8.06175*** 7.26098***	5.08240 4.37194
EMPSERV does not homogeneously cause MOBILE MOBILE does not homogeneously cause EMPSERV	6.27013*** 5.46104**	3.36847 2.67081	2.13503 5.54946***	−0.17594 2.85343
EMPSERV does not homogeneously cause TRAM TRAM does not homogeneously cause EMPSERV	7.21162*** 7.63315***	4.18029 4.54376	2.54499 1.36277	0.18779 −0.86111
MOBILE does not homogeneously cause ICT ICT does not homogeneously cause MOBILE	3.17187 6.59657***	0.69692 3.64994	7.83305*** 2.00758	4.87950 −0.28902
TRAM does not homogeneously cause ICT ICT does not homogeneously cause TRAM	3.35374 1.32101	0.85374 −0.89903	2.83679 3.63561	0.44668 1.15542
TRAM does not homogeneously cause MOBILE MOBILE does not homogeneously cause TRAM	4.77790** 3.75382	2.08176 1.19872	2.32233 3.47835	−0.00976 1.01589
Model 4				
WOM B does not homogeneously cause ICT ICT does not homogeneously cause WOM B	2.50117* 3.38829***	1.84126 3.04378	3.65957*** 1.73766	3.48008 0.83250
WOM B does not homogeneously cause MOBILE MOBILE does not homogeneously cause WOM B	3.12413** 3.12413	2.68571 1.64197	2.74893** 3.91468***	2.22560 3.83151
WOM B does not homogeneously cause TRAM TRAM does not homogeneously cause WOM B	0.89974 1.61484	−0.32955 0.63979	14.7466*** 3.05558**	18.7533 2.64803
MOBILE does not homogeneously cause ICT ICT does not homogeneously cause MOBILE	1.93063 5.86194***	1.06786 6.39693	4.91387*** 2.42558*	5.20796 1.78017
TRAM does not homogeneously cause ICT ICT does not homogeneously cause TRAM	1.03192 0.31335	−0.15038 −1.12443	1.68175 16.3853***	0.75548 21.0107
TRAM does not homogeneously cause MOBILE MOBILE does not homogeneously cause TRAM	3.88986*** 0.83399	3.72368 −0.41869	2.97102** 30.1509***	2.53155 39.9739

***, **, and * denote 1, 5, and 10% significant levels, respectively.

For the GCC countries, the results of Model 1 show four significant unidirectional causal relationships from total employment to mobile, from total employment to trademark, from ICT to mobile, and from trademark to mobile. Models 2 and 3 results show two significant unidirectional causal relationships between the variables at a 1% significant level. Finally, Model 4 revealed one bi-directional causal relationship between women's employment and ICT, and three significant unidirectional causal relationships from women's employment, ICT, and trademark to mobile.

For the advanced countries, Models 1–3 results show one significant bi-directional causal relationship between variables. Finally, Model 4 showed four bi-directional causal relationships between women's employment and mobile; women's employment and trademark, trademark, and mobile; and mobile and ICT. In addition to two significant unidirectional causal relationships from women's employment to ICT and from ICT to trademark.

The detected causal relationships indicate the significant future dynamics of all selected labor market indicators concerning analyzed variables (ICT, Mobile, and Tram) in GCCs and advanced countries.

## Conclusion and recommendations

Digitalization is a significant factor that influences labor market indicators, and it gains importance after the COVID-19 outbreak. The growing expansion of digital technologies and communication infrastructures in the daily lives of individuals and businesses needs a qualified labor force to work toward an inclusive digital transformation that strengthens the business systems.

This paper is an attempt to investigate the effect of digitalization on employment from different perspectives by comparing GCC countries and some selected advanced countries. Using an autoregressive distributed model for the period 2000–2020, the results of the Panel ARDL model show that for GCC countries, mobile subscriptions, and trademarks positively affected industry employment, while ICT has a significant negative impact on industry employment. There is a negative relationship between ICT and mobile subscriptions on the one hand, and services employment, on the other hand, these results confirm the findings of Wisskirchen (2017). Regarding the integration of women into the labor market, mobile subscriptions and trademark applications positively affect women's employment.

For the advanced countries group, the results show that the ICTs and mobile subscriptions are the main support for employment in the industrial and services sector. Regarding the long-term adjustment speed, all the results are significant for the developed countries, while the GCC countries suffer from disequilibrium in the long run and a slow adjustment toward the long-term equilibrium.

These results can be explained by the need of GCC countries to develop research and development to support the adoption of digitalization. Since the end of the COVID-19 outbreak, the governments of GCC countries become more aware of the importance of digitalization in the job market. Although the fast growth in the number of companies and startups in the region, the propagation of innovative digital technologies in the labor market is still very low. According to the report of Strategy and Middle East (2022), the share of big data companies per million inhabitants is between 0 and 2.5 compared to an average of 4.4 for advanced countries.

The recommendations provided by the research are to focus on three main areas in the GCC countries to limit the digital gap compared to the advanced countries. The first area is to focus on the development of the national labor force with advanced digital skills with training and companies' engagement to ensure a durable digital economy (Schilirò, 2021). The second area is to reinforce research and innovation through the review of the educational process to meet the new requirements of the job market. The third area is the localization of digital products. GCC countries should work on formulating strategies for technology transfer to reduce the imports of new technologies that will be costly for the government to train the labor force on them.

## Data availability statement

The datasets presented in this study can be found in online repositories. The names of the repository/repositories and accession number(s) can be found below: world bank database.

## Author contributions

JB is responsible for paper writing and model and data selection. FH is responsible for literature review. ME is responsible for the methodology review, model and data selection. All authors contributed to the article and approved the submitted version.

## Conflict of interest

The authors declare that the research was conducted in the absence of any commercial or financial relationships that could be construed as a potential conflict of interest.

## Publisher's note

All claims expressed in this article are solely those of the authors and do not necessarily represent those of their affiliated organizations, or those of the publisher, the editors and the reviewers. Any product that may be evaluated in this article, or claim that may be made by its manufacturer, is not guaranteed or endorsed by the publisher.

## Footnotes

^1^Gulf Cooperation Council countries include the Kingdom of Saudi Arabia, United Arab Emirates, Qatar, Kuwait, Oman, and Bahrain.
